# Mitochondrial oxidative phosphorylation controls cancer cell's life and death decisions upon exposure to MAPK inhibitors

**DOI:** 10.18632/oncotarget.7790

**Published:** 2016-02-29

**Authors:** Paola Corazao-Rozas, Pierre Guerreschi, Fanny André, Pierre-Elliott Gabert, Steve Lancel, Salim Dekiouk, Delphine Fontaine, Meryem Tardivel, Ariel Savina, Bruno Quesnel, Laurent Mortier, Philippe Marchetti, Jérome Kluza

**Affiliations:** ^1^ University Lille, Inserm, CHU Lille, UMR-S 1172 - JPArc - Centre de Recherche Jean-Pierre AUBERT Neurosciences et Cancer, Lille, France; ^2^ Institut pour la Recherche sur le Cancer de Lille (IRCL), Lille, France; ^3^ SIRIC OncoLille, Lille, France; ^4^ University Lille, Inserm, CHU Lille, Institut Pasteur de Lille, U1011- EGID, Lille, France; ^5^ Centre de Bio-Pathologie, Plate-forme de Biothérapie, Banque de Tissus, CHRU Lille, Lille, France; ^6^ Bioimaging Center, Lille Nord de France-Campus HU, Université de Lille 2, Lille, France; ^7^ Institut Roche, Boulogne-Billancourt, France

**Keywords:** vemurafenib, cobimetinib, BRAF, Ca2+ uptake, melanoma

## Abstract

Although MAPK pathway inhibitors are becoming a promising anticancer strategy, they are insufficient to fully eliminate cancer cells and their long-term efficacy is strikingly limited in patients with BRAF-mutant melanomas. It is well established that BRAF inhibitors (BRAFi) hamper glucose uptake before the apparition of cell death. Here, we show that BRAFi induce an extensive restructuring of mitochondria including an increase in mitochondrial activity and biogenesis associated with mitochondrial network remodeling. Furthermore, we report a close interaction between ER and mitochondria in melanoma exposed to BRAFi. This physical connection facilitates mitochondrial Ca2+ uptake after its release from the ER. Interestingly, Mfn2 silencing disrupts the ER–mitochondria interface, intensifies ER stress and exacerbates ER stress-induced apoptosis in cells exposed to BRAFi *in vitro* and *in vivo*. This mitochondrial control of ER stress-mediated cell death is similar in both BRAF- and NRAS-mutant melanoma cells exposed to MEK inhibitors. This evidence reinforces the relevance in combining MAPK pathway inhibitors with mitochondriotropic drugs to improve targeted therapies.

## INTRODUCTION

Mutant BRAF V600E inhibitors prolong both overall and progression-free survival, but their long-term efficacy is limited due to the emergence of treatment resistance [1]. Failure to achieve complete cancer cell death is a mechanism that may participate in the onset of acquired BRAFi treatment resistance [2]. As a result of incomplete eradication, some cells survive to BRAFi exposure, allowing drug-tolerant cells to accumulate subsequent genetic mutations that ultimately confer a resistant phenotype by reactivating the MAPK pathway or other survival pathways. It is necessary to understand the mechanisms underlying the way cancer cells adapt to survive BRAFi, in order to improve the effectiveness of BRAFi therapy.

It is largely admitted that BRAF mutations reprogram melanoma metabolism [3, 4]. Not surprisingly, BRAF inhibitors deeply alter the metabolism of melanoma cells. On the one hand, vemurafenib suppresses glucose uptake and glycolysis long before the onset of tumor growth arrest and cell death [5]. On the other hand, BRAFi stimulate mitochondrial activity resulting from the increase of mitochondrial biogenesis through PGC1a expression [6] and/or Drp-1 mediated mitochondrial hyperfusion [7]. In this context, we and others demonstrated that melanomas exposed to BRAFi rapidly became dependent on OXPHOS for survival as demonstrated by the high sensitivity of BRAFi-treated cells to the induction of apoptosis in response to several mitochondrial respiratory chain inhibitors [6, 8, 9]. This cell addiction to mitochondria was also described in BRAFi-resistant cell lines [8, 10, 11]. Thus, mitochondrial addiction constitutes an adaptive “survival” response to the inhibition of glucose metabolism [9, 12].

Two reports recently showed that BRAFi impair endoplasmic reticulum (ER) homeostasis and activate ER stress signaling pathway [13, 14]. Upon BRAFi exposure, ER stress induces cell death through Ca2+ release from ER lumen into cytosol [13] and/or direct binding between ER chaperone Grp78 and BRAFV600E [14]. Mitochondria and ER are two organelles which can physically interact with one another through contact sites formed by mitochondria-associated membranes (MAM) [15]. MAM, facilitating Ca2+ and lipid transfer between the two organelles, are characterized by the expression of specific proteins including mitochondrial Mitofusin-2, ER-mitochondria Ca2+ transfer Channels IP3R, mitochondrial porin VDAC and ER chaperone GRP78. ER-mitochondrial interactions regulate organelle function and metabolism. Previous studies evidenced that Ca2+ transfer from the ER to mitochondria in MAM could increase several parameters of mitochondrial bioenergetics and consecutively modulate the early phase of ER stress responses [16, 17]. Therefore, we hypothesized that mitochondrial metabolism could control the fate of cancer cells via the modulation of ER stress-mediated cell death. Our findings provide new insights into the mitochondrial protective role in BRAFi exposure bearing important implications for the development of therapeutic strategies.

## RESULTS

### Melanoma cells reprogram their metabolism towards mitochondrial OXPHOS to survive BRAFi exposure

**Figure 1:**
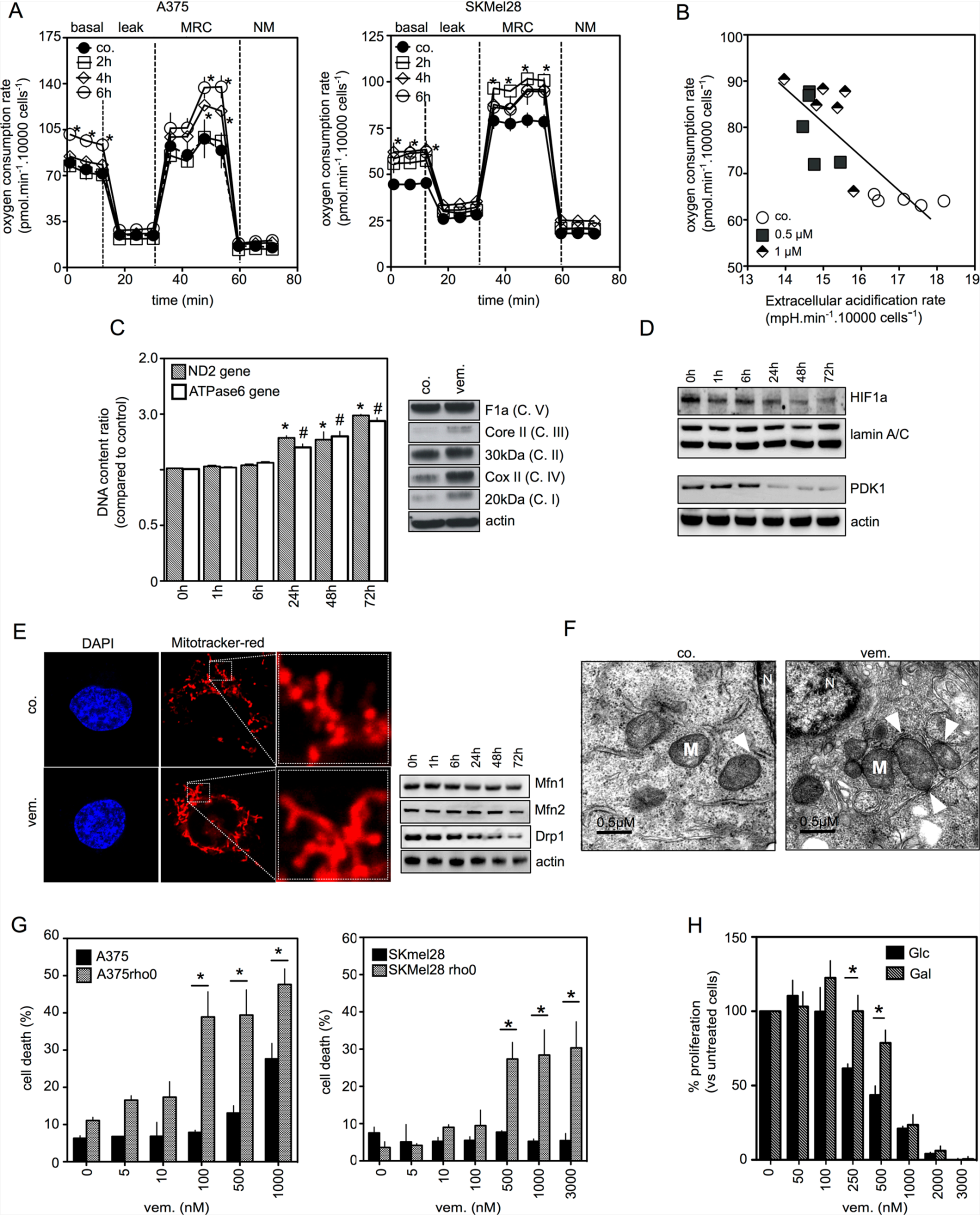
Coordinated changes in mitochondria of melanoma cells exposed to vemurafenib (**A**) Oxygen Consumption Rate in A375 *(left panel*) and SKMEL28 (*right panel)* treated with vemurafenib (0.5 μM) for 2, 4 and 6 hours. States of mitochondrial respiration are “basal” for basal respiration, “leak” for respiration after oligomycin exposure, “MRC” for maximal respiratory capacity and “NM” for non-mitochondrial respiration (*P < 0.05 compared to control); (**B**) Oxygen Consumption Rate and Extracellular Acidification Rate were measured simultaneously in A375 cells treated with vemurafenib for 6 hrs at 0.5 or 1 μM; (**C**) (*left panel)* Analysis of mitochondrial DNA copy number of A375 cells treated with vemurafenib (0.5 μM) for the indicated times (*n* = 3, *P < 0.05 compared to controls for ND2 gene and ^♯^*P* < 0.05 compared to controls for ATPase6 gene); (*right panel)* Immunoblotting of mitochondrial respiratory chain complex proteins in A375 treated or not with vemurafenib (0.5 μM) for 72 hrs; (**D**) (*upper)* Immunoblotting of nuclear HIF-1a expression in A375 cells treated by vemurafenib (0.5 μM) for the indicated times; (*bottom)* Immunoblotting of PDK1 expression in A375 cells treated as above; (**E**) (*left panel)* Confocal images of A375 cells stained with Mitotracker red that labels mitochondria (×630). Before staining, cells were untreated or treated with vemurafenib (0.5 μM) for 6 hrs (*right panel)*. Immunoblotting of Mfn1, Mfn2 and Drp1 in A375 cells treated by vemurafenib (0.5 μM) for the indicated times; (**F**) Transmission electron microscopic images of A375 melanoma cells untreated or treated with vemurafenib (0.5 μM) for 6 hrs (×67 000) (M: Mitochondria; N: Nucleus; arrows indicate ER/Mitochondria appositions); (**G**) A375 and SKMEL28 cells and respiratory-deficient A375rho0 and SKMEL28rho0 cells were exposed to vemurafenib at the indicated concentrations for 48 hrs (for A375/A375rho0) or 72 hrs (for SKMEL28/SKMEL28rho0) then cell viability was estimated by PI (*P < 0.05); (**H**) Glucose or galactose-growing A375 cells were exposed to vemurafenib at the indicated concentrations for 72 hrs and number of cells was estimated by counting (*P < 0.05, compared to respective control).

In BRAF mutant melanoma cell lines, A375 and SKMEL28, vemurafenib induced a dose-dependent increase in routine respiration and maximum respiratory capacity, two fundamental parameters of mitochondrial function, within six hours of exposure (Figure 1A and S1A). Concomitantly, vemurafenib reduced glucose uptake, glycolysis and the expression of the glucose transporter Slc2a3 mRNA (Figure S2A–S2C). When the extracellular acidification rate (ECAR), marker for glycolysis, decreased, oxygen consumption rate (OCR) increased (Figure 1B) confirming the shift from glycolytic metabolism towards mitochondrial OXPHOS. Accordingly, the hexokinase 2 inhibitor, 2-deoxyglucose, induced a strong inhibition of glycolysis and compensatory increase in OCR (Figure S2D).

Secondly, we explored the existence of other mitochondrial changes induced by BRAFi that could be associated with mitochondrial OXPHOS. Mitochondrial mass was significantly increased upon BRAFi exposure as evidenced by the enhancement of mitochondrial DNA content and the increased expression of several respiratory chain proteins (Figure 1C and S1B). We previously found that the HIF-1α/PDK axis was a major repressor of mitochondrial function in melanoma [18]. Similarly, HIF-1α and PDK1 were constitutively expressed in A375 and SKMEL28 cells and the level of expression of these proteins was reduced upon vemurafenib exposure (Figure 1D and S4A). Since the inhibition of PDK by dichloroacetate increases OXPHOS in A375 cells (Figure S1C), one can assume that the downregulation of the HIF-1/PDK axis could contribute to mitochondrial reprogramming observed in vemurafenib-treated cells. As observed by Serasinghe *et al.* [7], vemurafenib promoted the onset of a hyperfused mitochondrial network associated with the downregulation of Drp-1 protein expression (Figure 1E). No changes in the expression of mitochondrial fusion-related proteins Mfn1 and Mfn2 was observed. Moreover, vemurafenib exposure resulted in the subcellular redistribution of mitochondria to the nuclear periphery (Figure 1E and S1D, S4B). The perinuclear distribution of mitochondria was associated with close appositions of ER and mitochondria as evidenced via transmission electron microscopy (Figure 1F).

**Figure 2:**
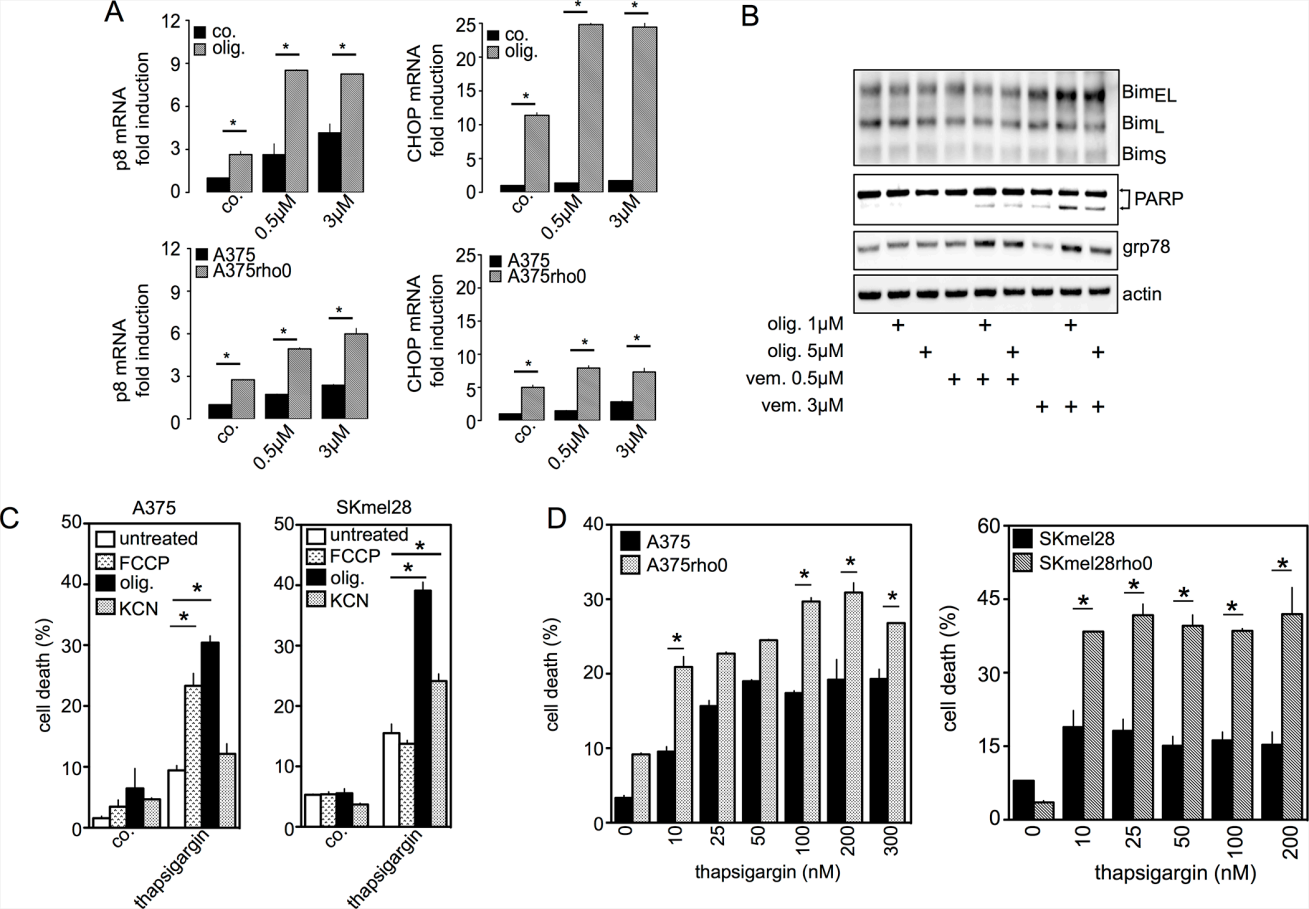
Inhibition of mitochondrial OXPHOS increases UPR signaling pathways and apoptotic cell death induced by vemurafenib (**A**) A375 cells were exposed to 0.5 μM or 3 μM vemurafenib for 24 hrs in the presence or absence of oligomycin (1 μM) *(upper panel).* A375 and respiratory-deficient A375rho0 cells were exposed to 0.5 μM or 3 μM vemurafenib for 24 hrs (*lower panel*). For both conditions p8 and CHOP mRNA were quantified (*n* = 3; *P < 0.05 compared to respective controls); (**B**) Immunoblotting of BIM, GRP78 and PARP expression in A375 cells treated with vemurafenib (0.5 μM and 3 μM) for 72 hrs. For the indicated condition, cells were previously incubated with oligomycin; (**C**) A375 (*left panel*) and SKMEL28 (*right panel*) cells were exposed to oligomycin (1 μM), FCCP (1 μM) or KCN (1 mM), then treated with thapsigargin (200 nM) for 48 hrs. Cell viability was estimated by PI (*P < 0.05 compared to thapsigargin treatment alone); (**D**) A375 and SKMEL28 and respiratory-deficient cells (A375rho0 and SKMEL28rho0) were exposed to thapsigargin at the indicated concentrations for 48 hrs and cell viability was estimated by PI (*P < 0.05 compared to rho0 cells).

As previously reported [8, 6], respiratory chain inhibitors increase BRAFi-induced cell death demonstrating the mitochondrial addiction of these cells. Consistent with these previous data, oligomycin enhances vemurafenib-induced cell death in A375 (Figure 2B and S1E) and in SKMEL28 cells (Figure S4C and S4D). Next, we validated the protective role of mitochondrial OXPHOS using the A375rho0 or SKMEL28rho0 cells, devoid of mitochondrial DNA and therefore free from residual OXPHOS function (Figure S3A and S3B). Thus, A375rho0 and SKMEL28rho0 cells were much more sensitive to the pro-apoptotic effect of vemurafenib than the parental cell lines (Figure 1G and S3C). Conversely, increasing cells' dependence on OXPHOS (*e.g.* culturing A375 cells in a galactose medium [19]) (Figure S1F) made them more resistant to the anti-melanoma effects of vemurafenib (Figure 1H). Our data indicate that BRAFi exposure can induce multifaceted mitochondrial adaptive responses that reduce treatment efficacy.

### The protective role of mitochondrial OXPHOS in response to BRAFi-induced ER stress

This mitochondrial reprogramming can be seen as a necessary mechanism to supply energy during glucose uptake inhibition [9]. Because apoptotic cell death induced by BRAFi is mediated by ER stress [13, 14], we hypothesized that mitochondrial reprogramming could also modulate ER stress and consecutively cell death induced by BRAFi. As previously described [14], vemurafenib-induced ER stress triggered the UPR signal transduction pathway characterized by the increased expression of the chaperone GRP78, activation of the PERK/eIF-2α pathway as evidenced by the abundance of P8, ATF4, ATF3, CHOP mRNA (Figure S5A–S5C). Ultimately, prolonged UPR activation by vemurafenib resulted in cell death by apoptosis (Figure S5C). Since mitochondria-deficient cells are sensitive to vemurafenib-mediated apoptosis (Figure 1), we evaluated to what extent the inhibition of mitochondrial OXPHOS could modify UPR activation, inciting cell death. Oligomycin drastically increased levels of p8 and CHOP mRNA in vemurafenib-treated A375 cells (Figure 2A). Consistent with the aforementioned results, A375rho and SKMEL28rho cells also exhibited higher levels of P8 and CHOP mRNA vs. untreated and vemurafenib-treated parental cells (Figure 2A) suggesting that OXPHOS inhibition leads to an overactivation of the PERK branch. In cancer cells, the combined therapy with vemurafenib and oligomycin substantially increased the expression of the ER-resident chaperone Grp78, the pro-apoptotic protein Bim and the cleaved fragment of PARP compared with vemurafenib treatment alone (Figure 2B). These findings suggest that upon BRAFi exposure, the oligomycin-induced inhibition of mitochondrial OXPHOS prolonged the activation of PERK/eIF2α, leading to a significant increase in apoptotic cell death. We assessed whether a similar behavior could be observed for other ER stress inducers such as thapsigargin. As observed with vemurafenib, thapsigargin triggered OXPHOS activation associated with a significant increase in ER-mitochondrial contacts at the perinuclear region (Figure S6C–S6D).

Once again, the inhibition of mitochondrial OXPHOS by FCCP, KCN or oligomycin increased thapsigargin-induced cell death in A375 and SKMEL28 cells (Figure 2C). Likewise, A375rho and SKMEL28-rho0 cells were more inclined to thapsigargin-induced cell death than parental cell lines (Figure 2D). Altogether these data indicate that mitochondrial OXPHOS regulates ER stress, UPR signaling and subsequent BRAFi-induced cell death.

### BRAFi increase the capacity of mitochondrial Ca2+ buffering in melanoma

**Figure 3:**
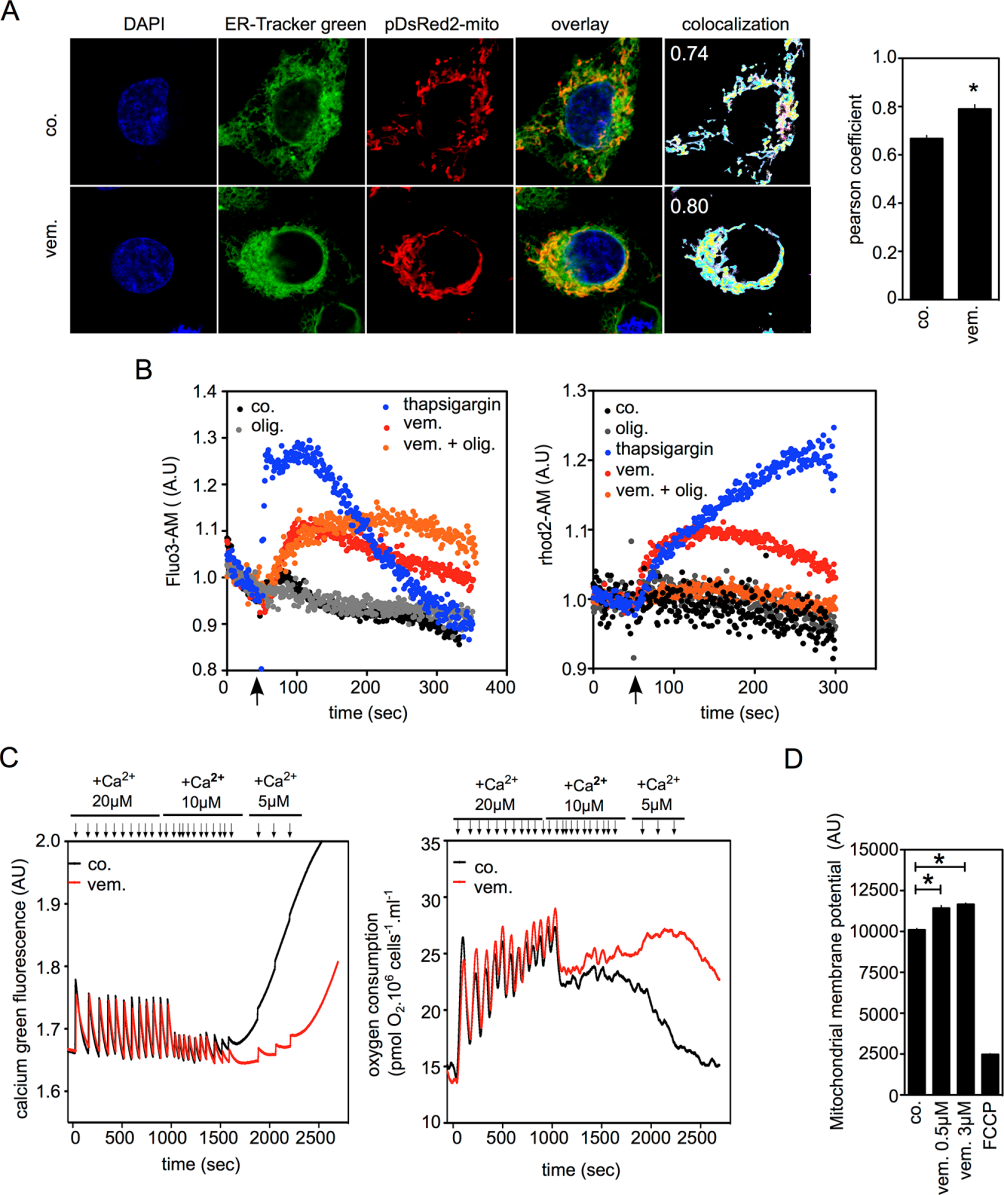
Vemurafenib increases Mitochondria/ER contacts and mitochondrial Ca2+ uptake (**A**) Confocal images of A375 cells stained with ER-tracker and pDsRed2-Mito labeled mitochondria. Cells were treated with vemurafenib (0.5 μM) or kept untreated for 6 hrs. (*right panel*) Quantitative analysis of ER-mitochondria co-localization were performed with Pearson's correlation coefficient [44] (*n* = 12; *P* < 0.05; *r* = −0.788); (**B**) Real-time monitoring of intracellular Ca2+ flux via flow cytometry within A375 cells treated with vemurafenib (50 μM) (black arrow), when indicated on the figure, cells were pre-incubated with oligomycin (1 μM). Cytosolic and mitochondrial Ca2+ were detected by staining with Fluo3-AM (*left panel*) and Rhod-2AM probes, respectively (*right panel*). Thapsigargin (25 nM) was used as positive control; (**C**) Simultaneous estimation of mitochondrial Ca2+ buffering capacity and oxygen consumption in permeabilized A375 cells following vemurafenib exposure. A375 cells (10^6^/ml) were exposed either to DMSO (0.1%) or to vemurafenib (3 μM). Sequential addition of Ca2+ (arrows) ultimately led to Ca2+ overload as indicated by the release of Ca2+ in the medium and reduction in mitochondrial respiration. Ca2+ concentration in the medium (*left panel*) and oxygen consumption (*right panel*) were measured using the O2K-oxygraph apparatus; (**D**) Measurements of mitochondrial membrane potential in A375 treated or not with vemurafenib (0.5 or 3 μM; 15 min).

It is established that ER-mitochondria contact sites play a critical role in Ca2+ signaling, thus we studied Ca2+ transfer from the ER to the mitochondria during BRAF inhibition (Figure 3). Consistent with TEM data (Figure 1F), staining for ER and mitochondria of vemurafenib-treated cells revealed similar spatial organization and contact sites between these organelles (Figure 3A). We used flow cytometric protocols to detect Ca2+ simultaneously in both cytosolic (Fluo-3AM) and mitochondrial (Rhod2-A) compartments (Figure 3B). Thapsigargin (inhibitor of Ca2+ reuptake into the ER) transiently increased the cytosolic Ca2+ concentration followed by mitochondrial Ca2+. Similarly, vemurafenib increased both cytosolic and mitochondrial Ca2+ concentrations. Interestingly, the oligomycin-induced inhibition of mitochondrial OXPHOS abolished mitochondrial Ca2+ uptake while maintaining high levels of Ca2+ in cytosol in vemurafenib-exposed cells. To validate vemurafenib-induced mitochondrial Ca2+ uptake capacities, mitochondrial Ca2+ accumulation (Figure 3C, left) and oxygen consumption (Figure 3C, right) were evaluated in permeabilized cells exposed to vemurafenib. Addition of Ca2+ led to a transient increase of Ca2+ in the medium followed by a rapid return to the initial level corresponding to the buffering ability of mitochondria to accumulate excess Ca2+. In parallel, oxygen consumption rates were elevated confirming the role of mitochondria in Ca2+ signaling (Figure 3C, right). Vemurafenib treatment significantly increased the buffering capacity of mitochondria and prevented the reduction of mitochondrial respiration due to Ca2+ overload. These data are in agreement with the increased mitochondrial membrane potential observed during the course of vemurafenib treatment (Figure 3D). Altogether, our results suggest that BRAFi enhance the number of contact sites between the ER and mitochondria, which would result in an increased Ca2+ flux between the ER and mitochondrion.

### Mfn2 modulates vemurafenib-induced ER stress responses

**Figure 4:**
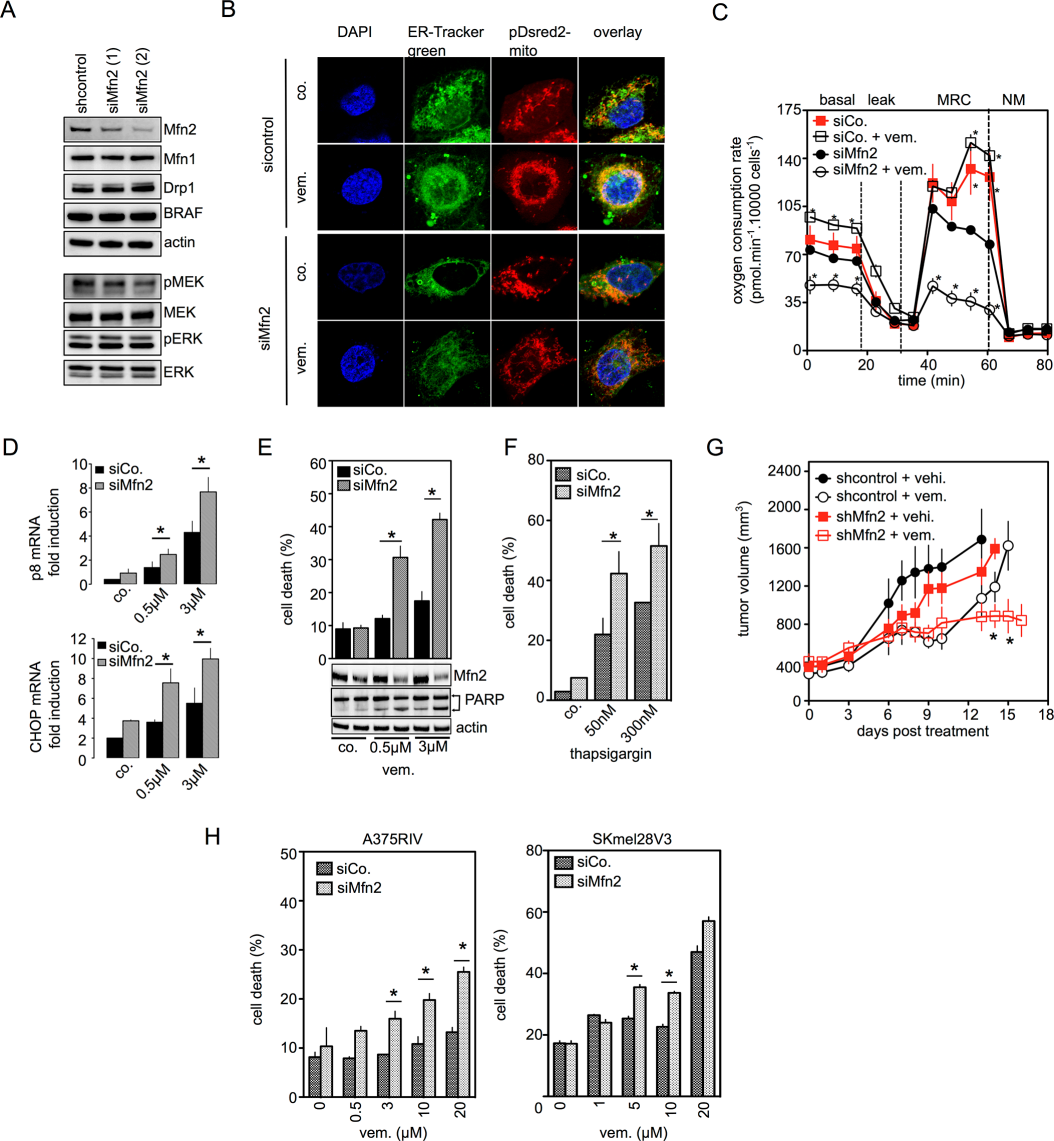
Mfn2 is involved in the mitochondrial protection against ER stress-related cell death induced by BRAFi (**A**) Immunoblotting of Mfn2, Mfn1, Drp1, BRAF, phospho-MEK and MEK, phospho-ERK and ERK in control siRNA or siMfn2 A375 cells; (**B**) Confocal images of siControl or siMfn2 A375 cells stained with ER-tracker and pDsRed2-Mito labeled mitochondria. Cells were untreated or treated with vemurafenib (0.5 μM) for 6 hrs; (**C**) Oxygen Consumption Rate in siControl or siMfn2 A375 cells treated with vemurafenib (0.5 μM) for 6 hrs. (**D**) Sicontrol or siMfn2 cells were exposed to vemurafenib (0.5 μM or 3 μM) for 24 hrs then p8 (*upper panel*) and CHOP (*lower panel*) mRNA were quantified (*n* = 3; *P < 0.05 compared to respective siControl group); (**E**) siControl or siMfn2 A375 cells were treated with vemurafenib at the indicated concentrations. Cell viability was estimated using PI (*upper panel*) and immunoblotting was performed for Mfn2 and PARP (*lower panel*); (**F**) SiControl or siMfn2 A375 cells were treated with thapsigargin at the indicated concentrations for 48 hrs. Cell viability was estimated by PI staining (*n* = 3, *P < 0.05 compared to respective controls); (**G**) A375 cells transfected with control shRNA or with Mfn2 shRNA were injected subcutaneously into SCID mice, which were divided into two separate treatment groups (vemurafenib or vehicle); (**H**) SiControl or siMfn2 vemurafenib-resistant A375RIV or SKMEL28V3 cells were treated with vemurafenib at the indicated concentration. Cell viability was estimated by PI staining (*n* = 3; *P < 0.05).

Mitofusin 2 (Mfn2) is a mitochondrial protein that was first involved in the mitochondrial fusion process. Mfn2 also constitutes a physical tether coupling the ER to mitochondria [15]. To unravel the role of Mfn2 in the protection against ER stress in BRAFi-induced apoptosis, we used knockdown of Mfn2 gene expression (Figure 4). Mfn2 knockdown does not affect other proteins involved in mitochondrial fusion/fission processes nor does it affect MAPK pathway activation (Figure 4A).

However, Mfn2 knockdown cells exhibited a more fragmented mitochondrial network vs. control siRNA cells. Interestingly, vemurafenib induced the redistribution of reticular and mitochondrial networks towards the perinuclear area as well as ER-mitochondria contact sites in control cells, but not in Mfn2 siRNA cells (Figure 4B). In addition, the absence of Mfn2 reduced mitochondrial respiration and vemurafenib does not increase OXPHOS in Mfn2 knockdown cells (Figure 4C). We further examined the consequences of Mfn2 knockdown on vemurafenib-induced ER stress responses. As seen in Figure 4D, vemurafenib increased p8 and CHOP mRNA abundance which were significantly higher in Mfn2 siRNA cells than in control cells. Next, we evaluated the effect of Mfn2 knockdown in apoptotic cell death induced by vemurafenib or thapsigargin. Compared with control siRNA cells, Mfn2 siRNA increased cell death in A375 and SKMEL28 cells treated with vemurafenib or thapsigargin (Figures 4E–4F and S4E**)**. In mouse xenograft models, Mfn2 knockdown did not alter tumor volume, whereas the absence of Mfn2 significantly prolonged the anti-melanoma effect of vemurafenib **(**Figure 4G**)**. Moreover, Mfn2 siRNA significantly increased vemurafenib-induced cell death in vemurafenib-resistant melanoma cell lines A375RIV and SKMEL28V3 (Figures 4H, S7A–S7B), which exhibited a high OXPHOS phenotype [8].

### Mitochondrial oxidative phosphorylation limits cell death induced by MEK inhibitors in MAPK-driven melanoma

**Figure 5:**
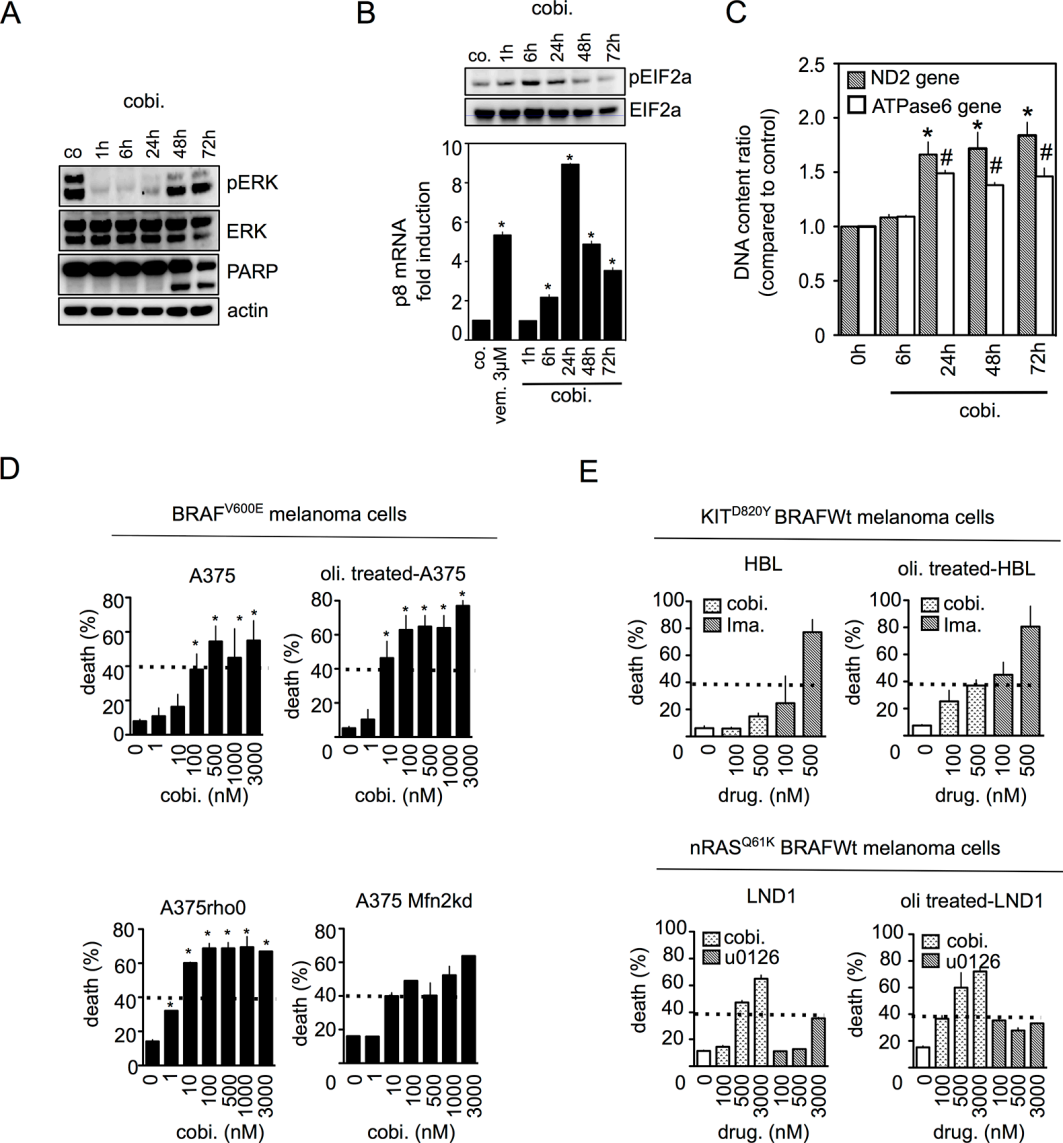
Mitochondrial OXPHOS limits cell death induced by MEK inhibitors in melanoma with constitutive MAPK activation (**A**) Immunoblotting of phospho-ERK, ERK and PARP in A375 cells treated with cobimetinib (100 nM) for the indicated times; (**B**) (*upper panel*) Immunoblotting of phospho-eIF2a and eIF2A in A375 cells treated as in (A); (*lower panel*) A375 were exposed to cobimetinib (100 nM) for the indicated times and p8 mRNA was quantified (*n* = 3; *P < 0.05 compared to controls); (**C**) Analysis of mitochondrial DNA copy number in cells treated with cobimetinib (100 nM) for the indicated times; (**D**) A375 cells, oligomycin-treated A375, siMfn2 A375 cells or respiratory-deficient A375rho0 cells were treated with cobimetinib at the indicated concentrations for 72 hrs and cell viability was estimated with PI staining (*n* = 3; *P < 0.05); (**E**) (*upper panel*) KIT^D820Y^ HBL or (*lower panel*) nRAS^Q61K^ LND1 cells were treated with cobimetinib at the indicated concentrations in the presence or absence of oligomycin (1 μM) for 72 hrs and cell viability was estimated with PI staining. KIT^D820Y^ HBL cells were also treated with imatinib as controls (*n* = 3; *P < 0.05).

Like vemurafenib, cobimetinib (MEK inhibitor) induced a transient decrease in phospho-ERK starting 1 hr after the beginning of exposure, leading to ER stress responses and mitochondrial mass enhancement in A375 cells (Figure 5A–5C). At 48 hrs, apoptotic cell death was clearly present in A375 cells treated with cobimetinib (Figure 5A). Upon cobimetinib exposure, the percentage of cell death was higher in A375rho0 cells, siMfn2 A375 cells and oligomycin-treated A375 cells than in parental A375 cells (Figure 5D) suggesting that mitochondrial OXPHOS also protected cells from MEK inhibitors-induced death. As shown in Figure 5E, cobimetinib was also able to induce cell death in others melanoma cells addicted to MAPK pathway activation such as KIT^D820Y^ mutant HBL cells or nRAS^Q61K^ mutant LND cells. Once again, inhibition of mitochondrial OXPHOS with oligomycin potentialized cobimetinib-induced cell death (Figure 5E).

## DISCUSSION

Recently, Verduzco *et al.* [20] proposed that two successive steps result in BRAFi-acquired resistance, an early metabolic reprogramming followed by subsequent mutation(s) promoting cell proliferation. Melanoma cells have to reprogram their metabolism essentially by increasing mitochondrial OXPHOS to compensate the suppressed glycolytic metabolism. Thus, different subsets of melanoma expressing constitutively PGC1a [6, 21] or expressing the H3K4-demethylase JARID1B (slow-cycling cells) [22] are more likely to survive metabolic stress. Multiple mechanisms could result in OXPHOS increase upon BRAF inhibition. These potential mechanisms include stimulation of mitochondrial biogenesis, down-regulation of the HIF/PDK axis, mitochondrial fusion and inhibition of glucose uptake and glycolysis (*i.e.* the Crabtree effect). We found that mitochondrial reprogramming that started after short-term exposure to vemurafenib, is a master regulator of cell fate. Indeed, blocking the metabolic switch towards mitochondrial OXPHOS dramatically increased BRAFi-induced apoptosis [11]. This described phenomenon is also found with MEK inhibitors in MAPK-driven melanoma with KIT or NRAS mutations [6, 7].

As demonstrated ([13] and Figure 2), BRAFi cause an acute ER stress response. Upon BRAFi exposure, Ca2+ is released from ER lumen to cytosol contributing to impaired ER activity (Figure 3 and [13]). Indeed, we observed in this context that Ca2+ released from the ER leads to its uptake to the mitochondria resulting in Ca2+ overload. Moreover vemurafenib cells exhibit an increase in mitochondrial membrane potential correlated with the enhancement of Ca2+ retention in mitochondria. It is widely accepted that the mitochondrial uptake of Ca2+ is passive, mainly driven by the negative mitochondrial potential generated by the respiratory chain through mitochondrial Ca2+ uniporter (MCU) [23–25]. All these data suggest that mitochondria actively participate in the uptake of ER-released Ca2+ after BRAF inhibition. A situation similar to the one described after depletion of intracellular Ca2+ stores induced by thapsigargin or other physiological stimuli (like Histamine or IP3) [26].

The role of Ca2+ within mitochondria is ambivalent. In low concentrations, Ca2+ accumulation in mitochondria stimulates OXPHOS and provides ATP necessary to support protein folding during ER stress [27]. High levels of Ca2+ within mitochondria lead to Ca2+ overload and triggers the opening of mitochondrial permeability transition pore leading to apoptosis [26]. Furthemore, BRAFi-induced mitochondrial reprogramming delays the onset of Ca2+ overload and reduced the appearance of ER stress-mediated cell death.

BRAFi increase ER-mitochondria contact sites that critically depend on the protein Mfn2. These results are also in agreement with previous data describing a decrease in ER/mitochondria contacts and the potentiation of the ER stress response in Mfn2 knockdown or KO cells [15, 28–31]. In this context, disruption of mitochondria-ER interaction could reduce the efficacy of mitochondrial Ca2+ uptake in response to Ca2+ release from the ER. Another possibility is that Mfn2 could reduce ER stress responses by physically interacting with PERK protein, the key ER stress sensor in the UPR [31]. Nevertheless, Filadi *et al.* [32] recently underlined that Mfn2 ablation increases close contacts between mitochondria and the ER and they suggested that reduced Ca2+ transfer between the ER and mitochondria could be due to the lower expression of the MCU in Mfn2 KO cells. Regardless of these considerations, our findings reveal the presence of physical and functional connections between mitochondria and ER, thereby allowing mitochondria to prevent ER-mediated cell death induced by BRAFi.

Experimental strategies combining MAPK pathway inhibitors and drugs targeting mitochondrial OXPHOS are currently being evaluated *in vitro* and in pre-clinical models [33, 34]. In combination therapy, the mitochondrial addiction induced by oncogenic kinase inhibitors sensitize cancer cells to mitochondria inhibition. This strategy reduces doses of mitochondrial inhibitors thereby decreasing toxicity to healthy tissues [34, 35]. The clinically-approved antidiabetic drugs, metformin or phenformin, are potent inhibitors of the mitochondrial complex I and thereby, display synergistic inhibition of cancer cell viability when combined with BRAFi [36]. Interestingly, our work offers new attractive targets, such as mitochondrial calcium channel, for the design of drugs that could be potentially combined with BRAFi [37]. Specific MCU modulators could be useful to increase cancer cell death in combination with BRAFi. MCU inhibitors could be associated with BRAFi to increase ER stress and cell death. Thus, inhibition of mitochondrial Ca2+ uptake by RU360 increases ER stress and cell death induced by gold nanoparticles [38]. Conversely, MCU activators could also be useful to promote mitochondrial Ca2+ overload, thus triggering the opening of the permeability transition pore and subsequent cell death.

These findings provide a rationale for targeting mitochondrial metabolism in this context and for further attempts to use new mitochondrial-targeting drugs in combination with MAPK pathway inhibitors.

## MATERIALS AND METHODS

Chemicals Reagents (Sigma-Aldrich) (unless otherwise stated), Vemurafenib (Roche, Paris, France) and U0126 SelleckChem (Euromedex, Souffelweyersheim, France).

### Cell lines

A375 and SKMEL28 human melanoma cell lines were purchased from the American Type Culture Collection. HBL and LND human melanoma cells were kindly provided by Professor G. Ghanem (Institut Bordet, Belgium). Original cells were analyzed using Short Tandem Repeat (STR) DNA profiling (IGNA, France), grown in bulk and were never passaged for more than 4 weeks. Vemurafenib-resistant cells (A375RIV and SKMEL28V3) were generated as previously described [8]. A375, A375RIV, LND and HBL cell lines were maintained in RPMI with 10% FCS and SKMEL28/SKMEL28V3 in DMEM with 10% FCS. All cell lines were periodically tested for mycoplasma contamination.

### Generation of rho0 cells

The procedure to generate rho0 cells was based on published protocols using ethidium bromide [39]. Two different sublines (A375rho0 and SKMEL28rho0) were derived and controlled for mtDNA depletion using specific PCR reactions as described in the PCR analysis section.

### Mfn2 RNA interference

For transient transfection, melanoma cells (18 × 10^5^ cells) were transfected with either 40pMol Mfn2-targeted siRNA (sc-43928, Santa Cruz or KH18422P, Qiagen) or with non-specific siRNA (sc-37007). Transfections were performed in Opti-MEM (Invitrogen) using Lipofectamine plus (Invitrogen) following the manufacturer's instructions. For stable Mfn2 knockdown, A375 melanoma cells were transfected under the same condition using different plasmids carrying respective shRNA or non-specific shRNA sequences (see Supplementary Material and Methods).

### Immunoblotting

Cell lysates and protein separation were prepared as previously described [40]. After blocking for 1hr in 10% milk in TBS-Tween buffer, membranes were probed with primary antibodies (See Supplementary Table S1). Actin or lamin A/C served as loading control for cytosolic or nuclear fraction, respectively. Horseradish peroxidase-conjugated secondary antibodies from Rockland Immunochemicals were used (1:2000) for 1 hr then detection was carried out by chemoluminescence.

### Microscopic imaging

For confocal microscopy, cells were grown on coverslips then stained with CMXROS (25 nM) or ER-tracker green (5 μM) and counterstained with DAPI (50 μg/ml). Indicated cell lines were transfected with a plasmid encoding mitochondrially-targeted red fluorescent protein (pDsRed2-Mito, Clontech Laboratories, 0.5 μg/200,000 cells) using Lipofectamine according to the manufacturer's protocol. Before the experiments, cells were seeded on 24-mm glass coverslip before microscopic analysis (Leica DMR).

### Transmission electron microscopy

Cells were prepared for transmission electron microscopy as described [41].

### Cytofluorometric analysis

Cell viability was evaluated via propidium iodide (PI) staining (5 μg/ml). Changes in mitochondrial mass were analyzed with dye Mitotracker green (100 nM, 30 min, 37°C). Cytosolic or mitochondrial Ca2+ quantifications were assessed with Fluo3-AM (2.5 μM, 30 min, RT) or with Rhod2-AM (2.5 μM, 30 min, 37°C). Fluorescences were analyzed on a FACSCanto II cytofluorometer (Beckton Dickinson). All Fluorescent probes were purchased from Life-Technologies.

### High resolution respirometry and Ca2+ assessment

Cells were suspended in Ca2+ uptake buffer [42] and cells (2 × 10^6^) were placed into O2K-oxygraph chambers (Oroboros). After digitonin permeabilization (10 μg/mL/million cells), 0.2 μM calcium-green (Life Technologies) was added and fluorescence intensity was measured by O2K-Fluo Led2-module. Then, rotenone (0.1 μM), succinate (10 mM) and ADP (2.5 mM) were added. Ca2+ pulses (20, 10 or 5 μM) were performed until mitochondrial Ca2+ release was detected by massive fluorescence increase.

### PCR analysis

Quantitative detection of mRNA was performed via real-time PCR using Lightcycler 480 detector (Roche Applied Science) and comparison was done with the Pfafll method [40]. Transcript levels were normalized to those of TBP ARNm. Sequencing primers used are listed in Supplementary Table S2. Quantitative detection of mtDNA was performed by real-time PCR using ND2 and ATP6 mitochondrial gene normalized with ATP synthase B nuclear gene [43].

### Assessment of oxygen consumption and glycolysis activity

Respiratory capacity and glycolytic activity of cells were performed with the XF24 Extracellular Flux Analyser (Seahorse Bioscience) [8]. To determine Oxygen Consumption Rate (OCR), cells were resuspended in DMEM (D5030, Sigma) with L-glutamine (2 mM) and D-glucose (10 mM) and the following drugs were added: 1 μM oligomycin, 0.25–0.5 μM FCCP, 1 μM rotenone and 1 μM antimycin A. To determine extracellular acidification rate, cells were resuspended in DMEM with L-glutamine (2 mM) and the following compounds were added: D-Glucose (10 mM), oligomycin (1 μM), and 2-DG (100 mM).

### *In vivo* study

All animal procedures were conducted according to institutional guidelines. Eight-week old, severe combined immunodeficient (SCID) female mice were injected, under isoflurane anesthesia, with 2 × 10^6^ control shRNA or Mfn2 shRNA A375 cells, mixed (1:1 volume) with BD Matrigel Basement Membrane Matrix. When tumors reached 100-250 mm^3^, two groups were randomly divided (*n* = 5) and mice were treated with either saline solution or vemurafenib (75 mg/kg/day, by oral gavage for 5days/week).

### Statistical analysis

Statistics were performed with GraphPad Prism^®^ v5.00 (GraphPad Software). Data are presented as mean ± SD of three independent experiments. The student's *t*-test was used to compare data sets with statistical significance at *P* < 0.05.

Abbreviations

ER: endoplasmic reticulum; OCR: oxygen consumption rate; ECAR: extracellular acidification rate; OXPHOS: mitochondrial oxidative phosphorylation; MAM: Mitochondrial associated membranes; PI Propidium Iodide; PERK: protein kinase R-like endoplasmic reticulum kinase.

## SUPPLEMENTARY MATERIALS FIGURES AND TABLES


