# Supplementary material for: Mitochondrial oxidative phosphorylation controls cancer cell's life and death decisions upon exposure to MAPK inhibitors
Source: Oncotarget. 2016 Feb 29;7(26):39473–85. doi: 10.18632/oncotarget.7790 (PMC5129946; doi:10.18632/oncotarget.7790)
Supplement: Supplementary file 1 [file oncotarget-07-39473-s001.pdf]

Mitochondrial oxidative phosphorylation controls cancer cell's life and death decisions upon exposure to MAPK inhibitors

Supplementary Materials

SUPPLEMENTARY MATERIAL AND METHODS

Clonogenic assay and proliferation

Cells (500/well) were seeded into 6-well plates and treated with vemurafenib (3 µM) in culture medium. After 2 weeks of culture, colonies were stained with crystal violet and digital images were taken.

Glucose measurements

Glucose was measured in the extracellular medium using a SYNCHRON LX20 Clinical system (Beckman Coulter, Fullerton, CA USA).

RNA interference

MFN2 knockdown have been obtained from plasmid furnished with SureSilencing shRNA Plasmid KH18422P (Qiagen, Courteboeuf, France).

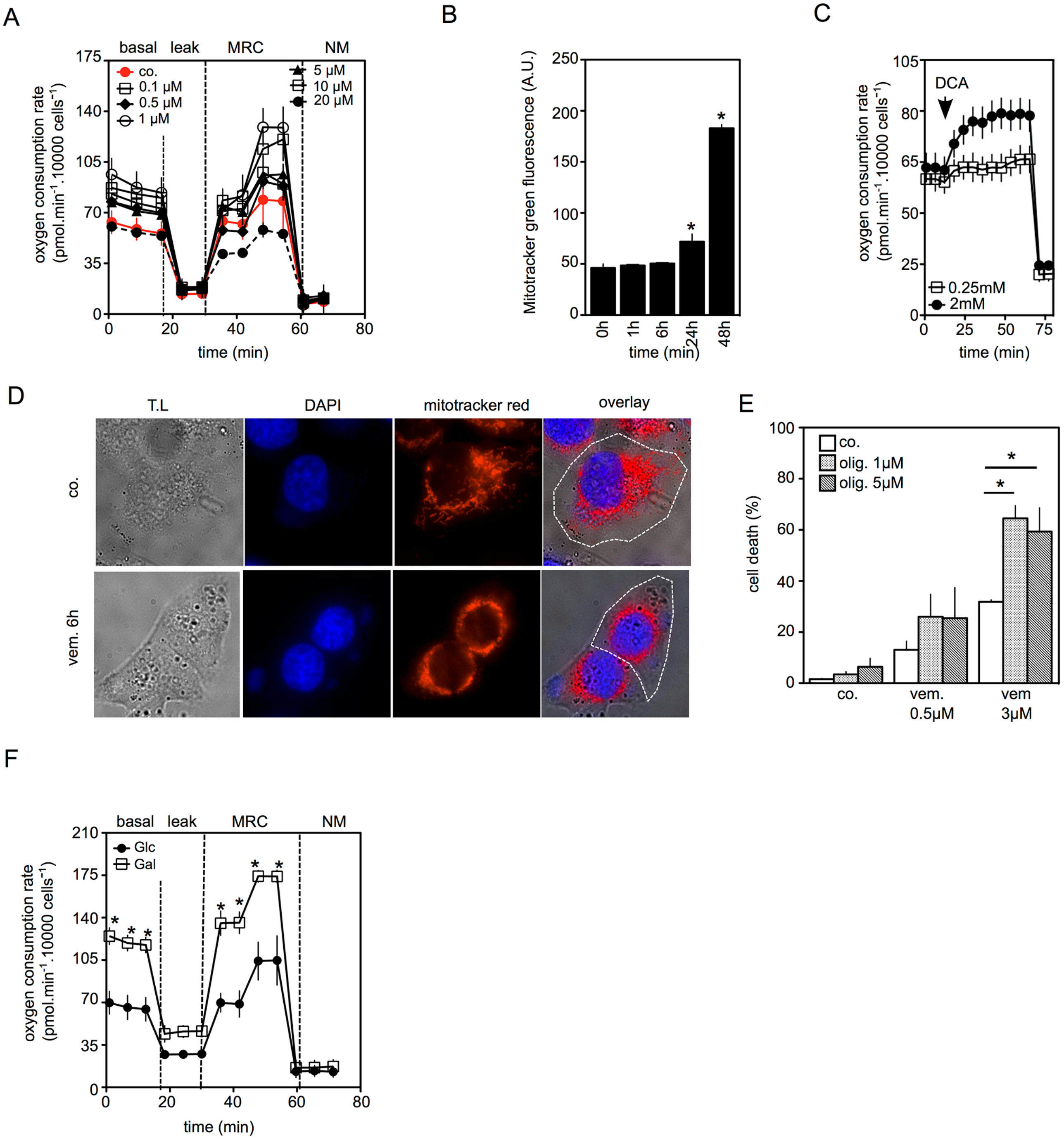

Supplementary Figure S1: (**A**) Oxygen consumption rate (OCR pmol/min/10000 cells) in A375 treated by vemurafenib for 6 hours at the indicated concentration. The different states of mitochondrial respiration are indicated as Figure 1A; (**B**) A375 cells have been treated with vemurafenib (0.5 µM) for the time indicated, then cells were labeled with the MTG fluorescent dye and relative mean fluorescence corresponding to mitochondrial mass has been evaluated by flow cytometry (*$P < 0.05$, compared to untreated condition); (**C**) Oxygen consumption rate (OCR pmol/min/10000 cells) in A375 cells. At the time indicated (black arrow), dichloroacetate (0.25 or 2 mM) is added; (**D**) Representative fluorescence microscopy images of A375 cells stained with Mitotracker red which localizes in mitochondria (Magnification, ×630). Before staining A375 cells have been treated with vemurafenib (0.5 µM) for 6 h or 24 h. Whole cell area is shown by white dotted line in overlay panel. Pictures are representative of 3 different experiments. (**E**) A375 cells were exposed to FoF1 ATP synthase inhibitor oligomycin A (1 and 5 µM), then treated with vemurafenib (0.5 µM or 3 µM) for 72 h. Cell viability was estimated by PI (mean ± SD of three independent experiments), *$P < 0.05$. (**F**) Oxygen consumption rate (OCR pmol/min/10000 cells) in A375 growing for 48 h in glucose or galactose medium. The different states of mitochondrial respiration are indicated as Figure 1A.

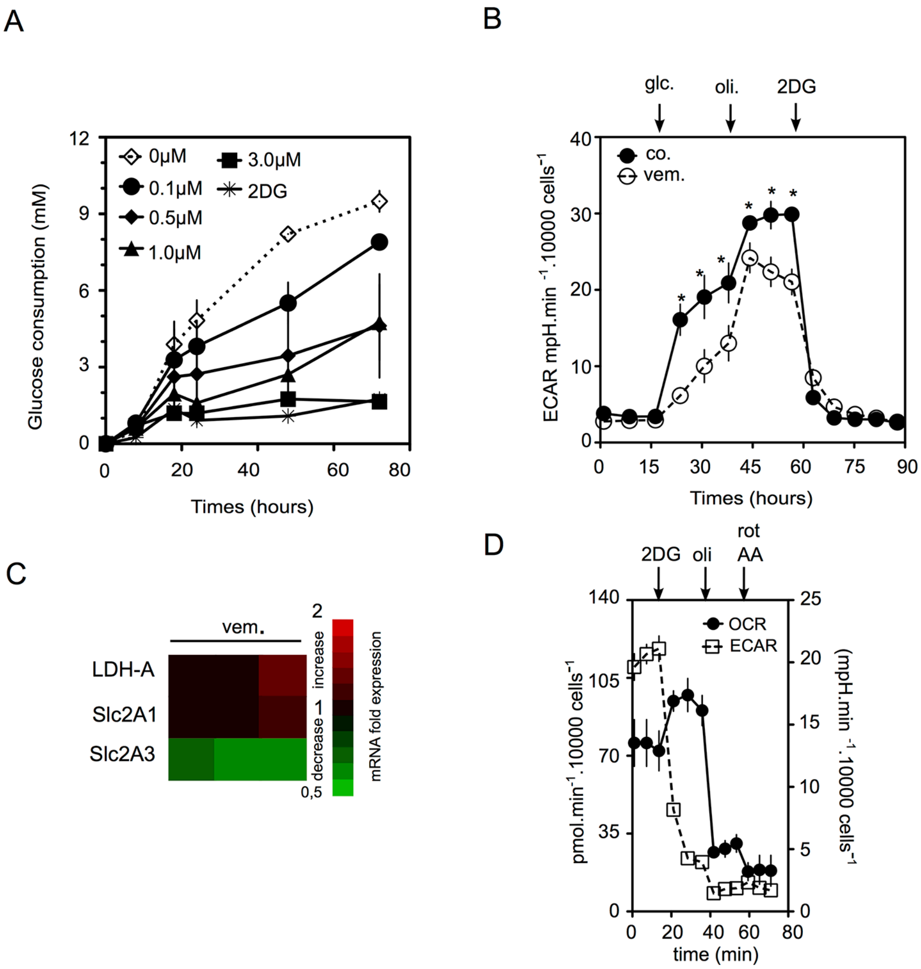

Supplementary Figure S2: (**A**) Glucose consumption has ben assessed in A375 cells treated at the indicated concentration with vemurafenib for 6, 12, 24, 48 and 72 h. 2-deoxyglucose (inhibitor of hexokinase 2) has been used as prototypic inhibitor of glucose uptake. (**B**) Extracellular acidification rate (ECAR) in A375 cells treated with vemurafenib (3 μM, 6 h); (**C**) quantitative PCR has been performed in A375 melanoma cells treated by vemurafenib (3 μM, 24 h) for LDH-A, Slc2A1 and Slc2A3 mRNA abundance; (**D**) Oxygen consumption rate (OCR) and extracellular acidification rate (ECAR) measured in A375 melanoma cells. At the time indicated, 2 deoxy-glucose (10 mM), oligomycin (1 μM) or rotenone (1 μM) / antimycin A (1 μM) were added.

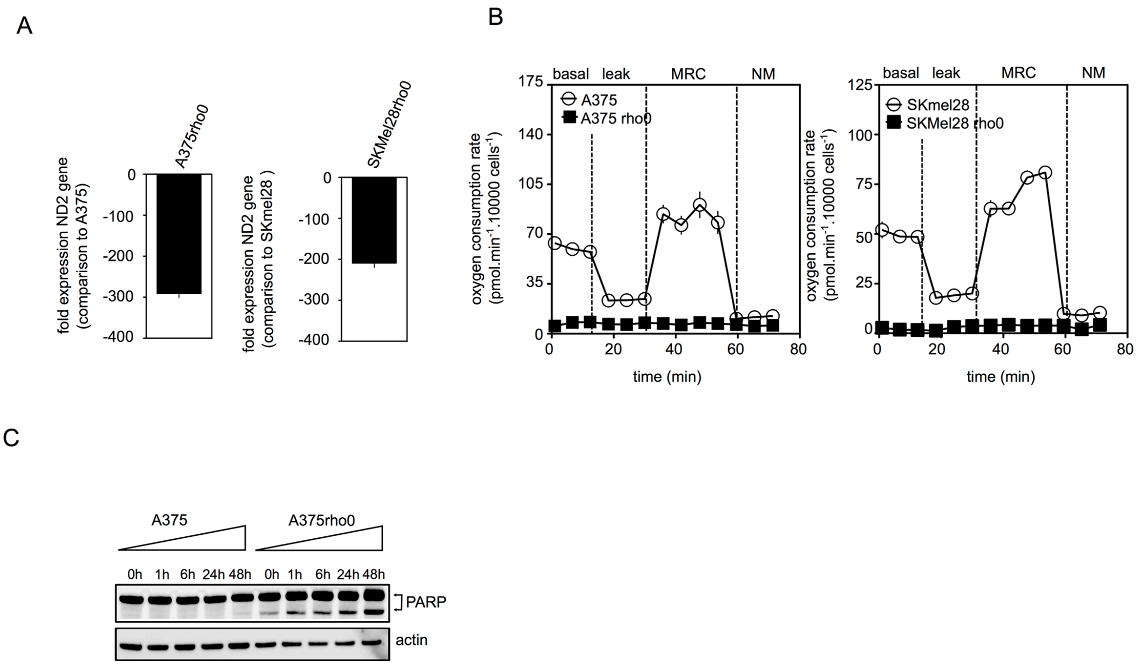

Supplementary Figure S3: (**A**) Relative evaluation of mitochondrial DNA copy number in A375 and respiratory-deficient A375rho0 (*left panel)* and SKMEL28 and respiratory-deficient SKMEL28rho0 (*right panel*). Total DNA was extracted and mitochondrial DNA copy number was measured by PCR amplification of ND2 mitochondrial DNA gene versus the nuclear ATP synthase gene (ATPsynβ) as standard control. Values are means ± SD; $n = 3$, $^{*}P < 0.05$ compared to control; (**B**) Oxygen consumption rate (OCR pmol/min/10000 cells) in A375 and respiratory-deficient A375rho0 cells. The different states of mitochondrial respiration are indicated as Figure 1A; (**C**) PARP expression in A375 cells treated with vemurafenib (0.5 µM) for the time indicated. Arrows correspond to 116 kDa and 85 kDa. Actin served as loading control.

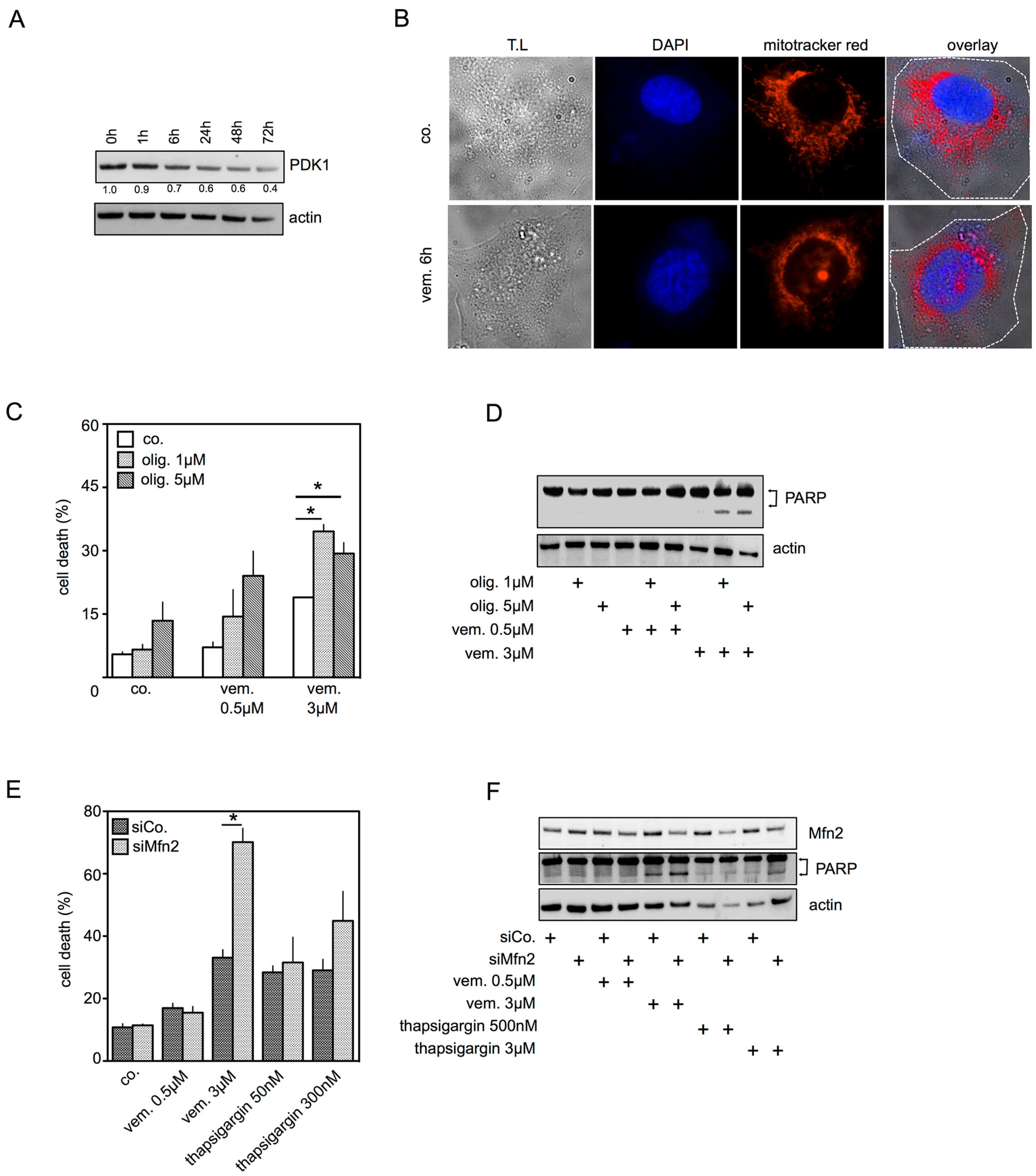

Supplementary Figure S4: (**A**) Immunoblotting of pyruvate dehydrogenase kinase 1 (PDK1) in SKMEL28 cells treated with vemurafenib (0.5 µM) for the time indicated. Actin served as loading control. Data are representative of 3 different experiments. (**B**) Representative fluorescence microscopy images of SKMEL28 cells stained with Mitotracker red which localizes in mitochondria (Magnification, ×630). Before staining SKMEL28 cells have been treated with vemurafenib (0.5 µM) for 6 h. Whole cell area is shown by white dotted line in overlay panel. Pictures are representative of 3 different experiments. (**C**) SKMEL28 cells were exposed to FoF1 ATP synthase inhibitor oligomycin A (1 and 5 µM), then treated with vemurafenib (0.5 µM or 3 µM) for 72 h. Cell viability was estimated by PI (mean ± SD of three independent experiments), $^{*}P < 0.05$. (**D**) Immunoblotting of PARP expression (arrows correspond to 116 kDa and 85 kDa) in SKMEL28 cells treated with vemurafenib (0.5 µM and 3 µM) for 72 h. For the indicated condition, cells have been prior incubated with oligomycin A (1 or 5 µM). Actin served as loading control. Data are representative of 3 different experiments; (**E**) siControl or SiMfn2 SKMEL28 cells were treated with vemurafenib or thapsigargin at the indicated concentration. Cell viability was estimated by PI (*left panel*) and immunoblotting was performed for Mfn2 and PARP (*right panel*). Actin served as loading control. Data are representative of 3 different experiments.

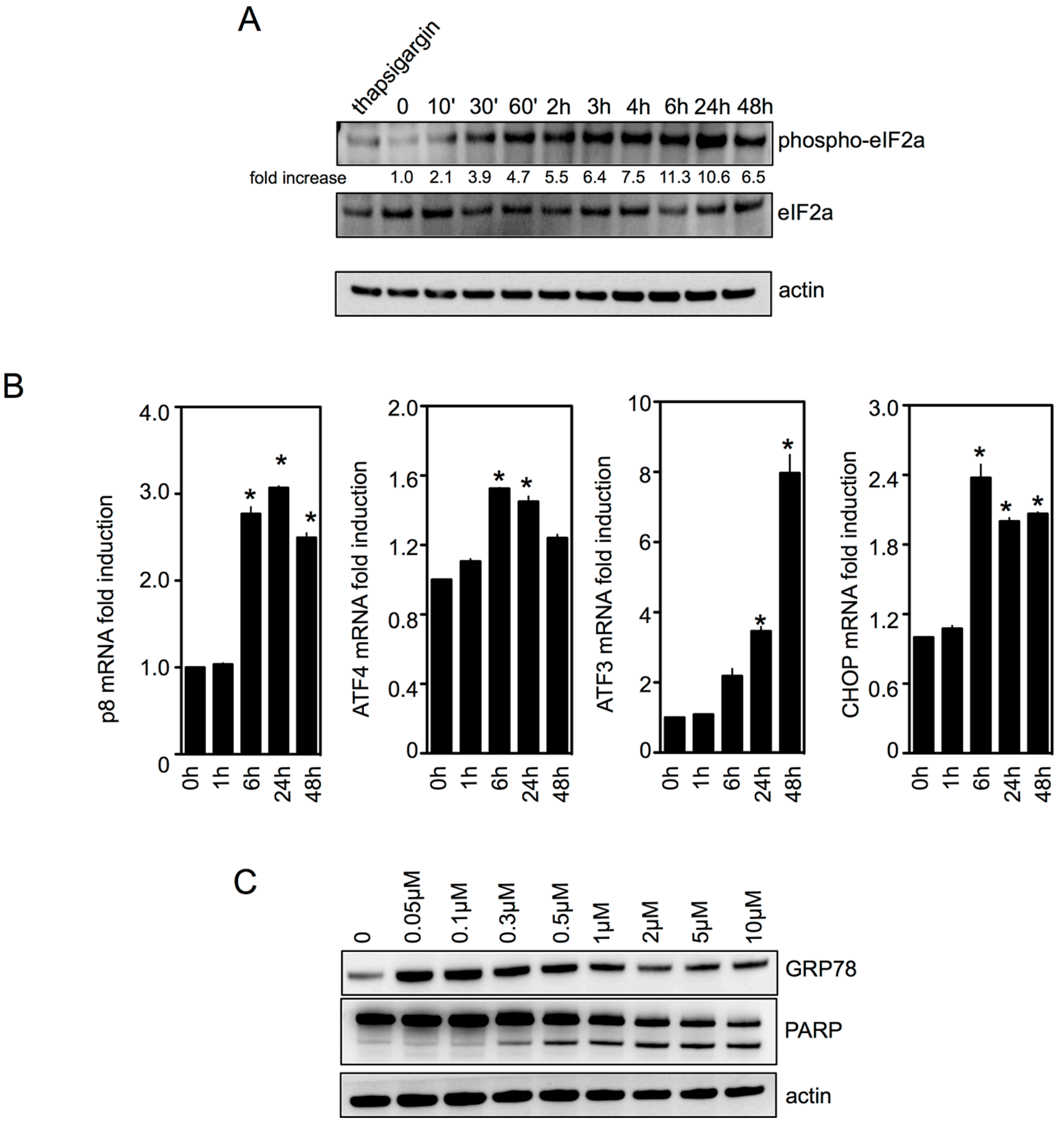

Supplementary Figure S5: (**A**) Immunoblotting of phospho-eIF2a and eIF2a in A375 cells treated with vemurafenib (0.5 µM) at the indicated time. Actin served as loading control. Densitometric values of proteins normalized on actin expression are expressed. (**B**) A375 were exposed to 0.5 µM vemurafenib at the indicated time then total RNA were subjected to quantitative RTPCR to quantify p8, ATF4, ATF3 and CHOP mRNA abundance. Results are mean ± SD ($n = 3$; $^*P < 0.05$ compared to control (0 h)); (**C**) Immunoblotting PARP expression and glucose related protein grp78 in A375 cells treated with vemurafenib at the concentration indicated for 72 h. Actin served as loading control.

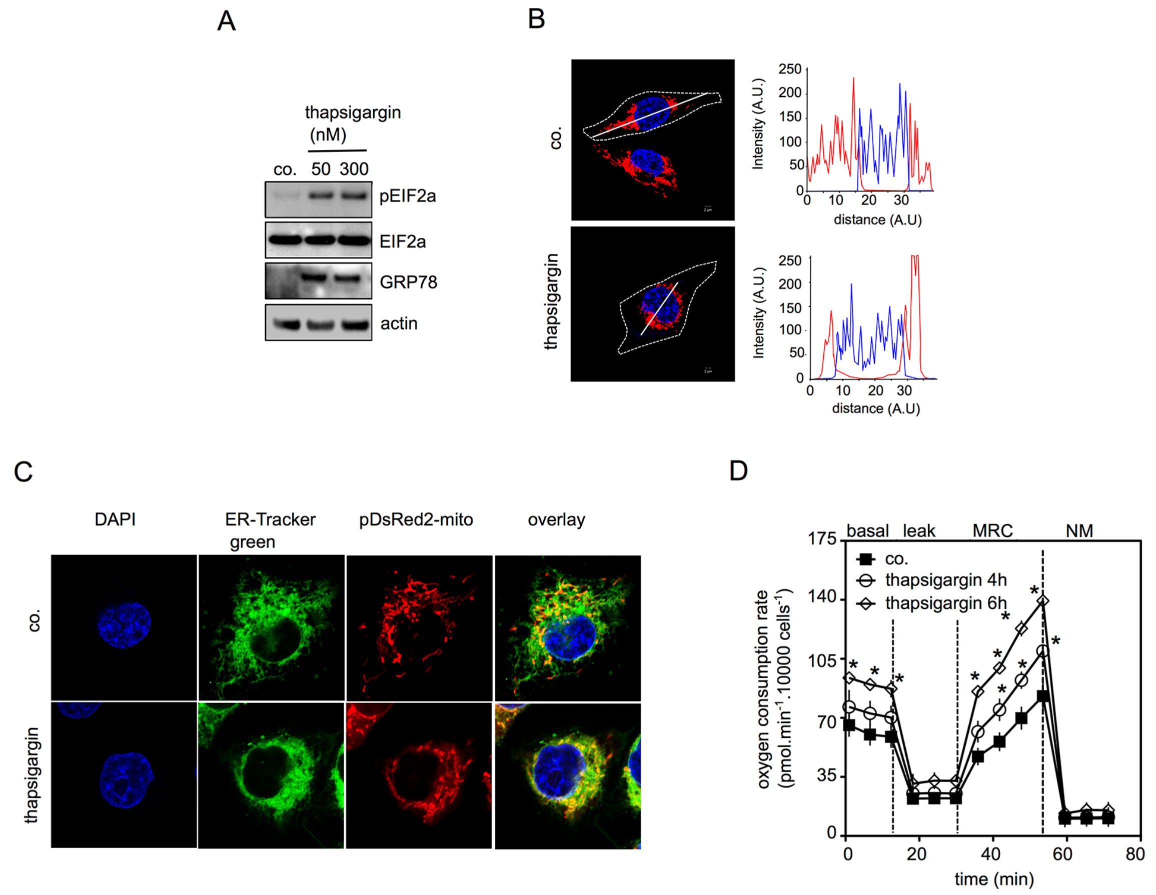

Supplementary Figure S6: (**A**) Immunoblotting of phospho-eIF2a and eIF2a, glucose related protein GRP78 in A375 cells treated with thapsigargin (50 nM or 300 nM) for 6 h. Actin served as loading control; (**B**) Representative confocal images of A375 cells stained with DAPI (blue) and Mitotracker red-labelled mitochondria (red). Cells have been untreated or treated with thapsigargin (300 nM) for 6 h. Intensity of blue fluorescence (nucleus and red fluorescence (Mitochondria) have been assessed in respective histograms; (**C**) Representative confocal images of A375 cells stained with ER-tracker (green) and pDsRed2mito labeled mitochondria (red). Cells have been untreated or treated with thapsigargin (300 nM) for 6 h; (**D**) Oxygen consumption rate (OCR pmol/min/10000 cells) in A375 treated or not with thapsigargin 300 nM for 4 and 6 h. The different states of mitochondrial respiration are indicated as Figure 1A.

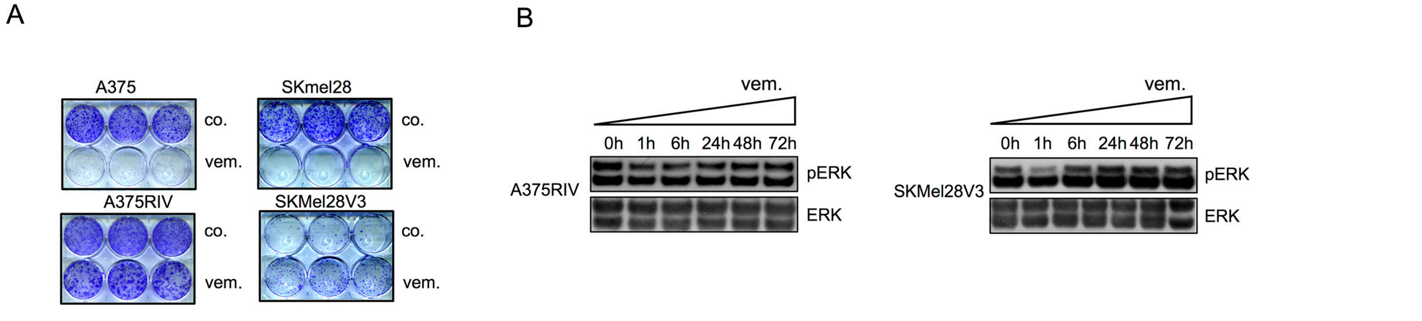

Supplementary Figure S7: (**A**) Colony-forming ability of A375 and SKMEL28 and respective vemurafenib-resistant melanoma cells (A375RIV or SKMEL28V3) treated or not with vemurafenib 3 µM. After two weeks colonies were stained with crystal violet. Data are representative of 3 different experiments; (**B**) Immunoblotting of phospho-ERK and ERK in vemurafenib-resistant A375RIV and SKMEL28 treated with vemurafenib (0.5 µM) for the time indicated.

Supplementary Table S1: List of antibodies used

Bim (1 :1,000, Santa Cruz Biotechnology inc., N-20), (**Ser51) phospho-eIF2A** (1 :1,000, Cell Signaling Technology Inc., Denvers, MA, #9721), **eIF2A** (1 :1,000, Cell Signaling Technology Inc., Denvers, MA, #9722), **Mfn2** (1 :1,000, Abcam, Ab56889), **PARP** (1 :1,000, Santa Cruz Biotechnology inc., H-250), **Actin** (1 :1,000, Santa Cruz Biotechnology inc., C-4). **OXPHOS complexes** monoclonal antibodies MS604/DO522) from MitoSciences. **p44/42 MAPK (Erk1/2)** (1:1000, Cell Signaling Technology Inc., Denvers, MA, #9102), **phospho-p44/42 MAPK (Erk1/2)** (1:1000, Cell Signalling Technology Inc., Denvers, MA,#9106), **HIF-1a** (1:1000, Santa Cruz Biotechnology inc., H-206), **PDK1** (1 :1,000, Abcam, Ab110335), **DRP-1** (1:1000, Santa Cruz Biotechnology inc., H-300), Grp78 (1:1000, Abcam, Ab21685), **Lamin A/C** (1:1000, Santa Cruz Biotechnology inc., 636), **RAF-B** (1 :1,000, Santa Cruz Biotechnology inc., F-7), **P-MEK1/2** (Ser217/221) (1:1000, Cell Signaling Technology Inc., Denvers, MA, #9121), **MEK1/2** (1:1000, Cell Signaling Technology Inc., Denvers, MA, #9122), **GRP78** (1 :1,000, Abcam, Ab21685).

Supplementary Table S2: List of PCR primers used

ATP synthaseB sense 5′-CTGACTGTGGCTGAATACTT-3′ and antisense 5′- CCCTTCTTGGTAGTGGTAAT-3′, **ND2** sense 5′-CTAGCCCCCATCTCAATCATA-3′ and antisense 5′- GAATGCGGTAGTAGTTAGGAT-3′ and **ATPase 6** sense 5′-CCTAGCCCACTTCTTACCACA-3′ and antisense 5′- GCTTGGATTAAGGCGACAG-3′ and **TBP** sense 5′-CCC-GAAACGCCGAATATAATCC-3′ and antisense 5′-GACTGTTCTTCACTCTTGGCTC-3′ and **p8** sense 5′-GCAG-CAGCTTCTCTCTTGGT-3′ and antisense 5′-CAGCCTGGATGAATCTGACC-3′ and **CHOP (DDIT3)** sense 5′-TG-GATCAGTCTGGAAAAGCA-3′ and antisense 5′- AGCCAAAATCAGAGCTGGAA-3′ and **ATF4** sens 5′-GAAGGT-CATCTGGCATGGTT-3′ and antisense 5′-AGTCCCTCCAACAACAGCAA-3′ and **CHOP** sens 5′-TGGATCAGTCTG-GAAAAGCA-3′ and antisense 5′-AGCCAAAATCAGAGCTGGAA-3′ and **ATF3** sens 5′- ACTTCCGAGGCAGAGA-CCTG-3′ and antisense 5′-GGCCAGACAAACAGCCC-3′.
